# Supplementary material for: Nurses’ Performance on the Genomic Nursing Inventory: A Cross-Sectional Study in Crete, Greece
Source: Nurs Rep. 2025 Mar 31;15(4):121. doi: 10.3390/nursrep15040121 (PMC12029944; doi:10.3390/nursrep15040121)
Supplement: Supplementary file 1 [file nursrep-15-00121-s001.zip › nursrep-3501858-supplementary.pdf]

Table S1. Contribution of each item to the GNCI scale.

	Cronbach's Alpha if Item Deleted
Q1	0.611
Q2	0.611
Q3	0.610
Q4	0.615
Q5	0.615
Q6	0.619
Q7	0.609
Q8	0.616
Q9	0.623
Q10	0.611
Q11	0.618
Q12	0.608
Q13	0.610
Q14	0.613
Q15	0.612
Q16	0.620
Q17	0.612
Q18	0.613
Q19	0.612
Q20	0.605
Q21	0.610
Q22	0.613
Q23	0.613
Q24	0.621
Q25	0.616
Q26	0.612
Q27	0.619
Q28	0.609
Q29	0.613
Q30	0.619
Q31	0.617
